# High Carriage of Extended-Spectrum, Beta Lactamase-Producing, and Colistin-Resistant Enterobacteriaceae in Tibetan Outpatients with Diarrhea

**DOI:** 10.3390/antibiotics11040508

**Published:** 2022-04-11

**Authors:** Zhe Li, Jiaqi Li, Jiaqi Liu, Yao Peng, Zhenpeng Li, Mengyu Wang, Ge Zhang, Geruo Qu, Jingyun Zhang, Xiuping Fu, Xia Chen, Ciren Dunzhu, Shan Lu, Xin Lu, Jialiang Xu, Biao Kan

**Affiliations:** 1 State Key Laboratory of Infectious Disease Prevention and Control, National Institute for Communicable Disease Control and Prevention, Chinese Center for Disease Control and Prevention, Beijing 102206, China; lizhe900504@hotmail.com (Z.L.); lijiaqi_19951122@163.com (J.L.); liujiaqi19990116@126.com (J.L.); pengyao@icdc.cn (Y.P.); lizhenpeng@icdc.cn (Z.L.); mengyu_w0110@163.com (M.W.); zg314706@163.com (G.Z.); qugeruo@163.com (G.Q.); zhangjingyun@icdc.cn (J.Z.); fuxiuping@icdc.cn (X.F.); chenxia@icdc.cn (X.C.); lushan@icdc.cn (S.L.); 2 School of Light Industry, Beijing Technology and Business University, Beijing 100048, China; 3 Tibet Center for Disease Control and Prevention, Lhasa 850000, China; cirendunzhuok@126.com

**Keywords:** antibiotic-resistant bacteria, antibiotic-resistance gene, diarrhea, extended-spectrum beta lactamase, colistin resistance

## Abstract

Antibiotic-resistant bacteria (ARB) and antibiotic-resistance genes (ARGs) have been detected in human-impacted habitats, especially in densely populated cities. The Qinghai–Tibet Plateau is located far from the heavily populated regions of China, and Tibetan residents have distinct dietary habits and gut microbes. Antibiotic-resistance monitoring in the Tibetan population is rare. Here, we collected stool samples from Tibetan outpatients with diarrhea. From 59 samples, 48 antibiotic-resistant Enterobacteriaceae isolates were obtained, including 19 extended-spectrum beta lactamase (ESBL)-producing isolates from 16 patients and 29 polymyxin-resistant isolates from 22 patients. Either ESBL or *mcr* genes were found in 17 *Escherichia coli* isolates, approximately 58.8% of which were multidrug-resistant, and ten incompatible plasmid types were found. The gene *bla*_CTX-M_ was a common genotype in the ESBL-producing *E. coli* isolates. Four *E. coli* isolates contained *mcr-1*. The same *mcr-1*-carrying plasmid was found in distinct *E. coli* isolates obtained from the same sample, thus confirming horizontal transmission of *mcr-1* between bacteria. Genomic clustering of *E. coli* isolates obtained from Lhasa, with strains from other regions providing evidence of clone spreading. Our results reveal a strong presence of ARB and ARGs in Tibetan outpatients with diarrhea, implying that ARB and ARGs should be monitored in the Tibetan population.

## 1. Introduction

Antibiotic resistance (AR) is a global health concern [1]. Humans (including healthy individuals and patients), wildlife, companion animals, livestock, fruits, vegetables, water, and soil are all directly or indirectly exposed to antibiotics, due to clinical or agricultural use or contamination [2]. This exposure threatens the effectiveness of antibiotics for treating and preventing bacterial infections [3]. One Health approaches focus on antibiotic-resistant bacteria (ARB) both as pathogens and as antibiotic-resistance gene (ARG) donors. Through food, transportation, and trade, the flow of ARB and transmission of ARG from the environment to animals and then to clinics and vice-versa is fairly common [4]. On a worldwide scale, the rapid emergence and dissemination of ARB and ARGs occurs in cities with high population densities, in villages with developed agricultural breeding, and in fields far from human activity.

The Qinghai–Tibet Plateau is known as the “Third Pole” and, with an average altitude of 4500 m above sea level, it has one of the highest elevations among inhabited areas worldwide. Its indigenous people have a relatively unusual lifestyle, especially in their dietary culture [5]. Additionally, significant differences exist in the gut microbiotas of the local Tibetan and Han populations [6]. The environment of the Qinghai–Tibet Plateau is unique, and the indigenous bacteria have rarely been exposed to anthropogenic antibiotics. The occurrence and prevalence of AR in this region remains poorly studied [7]. The Tibet Autonomous Region of China is located on the Qinghai–Tibet Plateau, far from densely populated regions. Compared with urban regions, the Tibetan Plateau has a low capacity for ARG selection and low carriage of ARGs by mobile genetic elements [8]. However, some cities in Tibet, such as Lhasa, have been modernized and have many economic and social exchanges with other parts of China through trade and travel. Recent studies found that *Escherichia coli* isolated from yaks in Tibet had a high rate of multidrug-resistance [9]. Monitoring ARB and ARG levels in Tibetan residents to track the spread of AR may help determine the risk of AR and improve understanding of AR. However, Tibet currently has limited surveillance of the drug-resistant bacteria among its residents in either the healthy individuals or the outpatients with diarrhea.

Multidrug-resistant (MDR) Gram-negative bacteria, such as extended-spectrum beta lactamase (ESBL)-producing Enterobacteriaceae and *mcr*-harboring Enterobacteriaceae, have attracted extensive attention from researchers. The *bla*_ESBL_s in Enterobacteriaceae leads to delays in the effective treatment of these MDR microorganisms, leading to higher rates of infection-related mortality, longer hospitalizations, and higher medical costs [10,11,12]. Owing to the limited treatment options for infection by ESBL-producing bacteria, the spread of these bacteria has become a major public health issue [13]. Colistin is often used as the last line of defense for treating MDR bacteria [14]. However, the discovery of *mcr-1* in plasmids showed that polymyxin resistance can be transferred horizontally between bacteria [15]. ESBL-producing Enterobacteriaceae and *mcr-1*-harboring Enterobacteriaceae have been found worldwide, leaving humans to face the risk of having no effective medications against bacteria.

In this study, we collected fecal samples from Tibetan outpatients with diarrhea in Lhasa. We screened for ARB (including ESBL-producing, colistin-resistant, and carbapenem-resistant bacteria) using antibiotic resistance plates and screened for ARGs via PCR. *E. coli* was used as an indicator organism and sequenced. The Tibetans in this study carried MDR bacteria, ESBL-producing *E. coli*, and *mcr*-carrying *E. coli*, as well as MDR strains from other regions, evidencing the potential threat that ARB and ARGs pose to public health.

## 2. Materials and Methods

### 2.1. Sample Collection, Bacterial Isolates, and Isolate Characterization

From August to October 2017, one fecal sample was collected from each of 59 Tibetan outpatients with diarrhea who resided primarily in Lhasa, Tibet and visited one of four sentinel hospitals in Lhasa. Stool specimens were collected with disposable stool collectors filled with Cary–Blair medium (Oxoid, Basingstoke, UK). All samples were screened for five pathotypes of diarrheagenic *Escherichia coli* (DEC): enteroaggregative *E. coli* (EAEC), enteropathogenic *E. coli* (EPEC), enterotoxigenic *E. coli* (ETEC), Shiga toxin-producing *E. coli* (STEC), and enteroinvasive *E. coli* (EIEC). The fecal samples were streaked on MacConkey agar to isolate DEC. To identify DEC isolates, suspected *E. coli* colonies on the MacConkey agar were selected and screened via real-time PCR [16]. The obtained DEC isolates, as well as all 59 stool samples, were then screened for antimicrobial-resistant Enterobacteriaceae and other Gram-negative bacteria by attempting to grow them on three chromogenic media, CHROMagar ESBL, Col-apse, and SuperCARBA (CHROMagar, Paris, France), to isolate ESBL-producing, colistin-resistant, and carbapenem-resistant bacteria, respectively. Colonies of different colors and shapes were selected from each plate. A matrix-assisted laser desorption/ionization time of flight mass spectrometry (MALDI-TOF MS) system (Autobio, Zhengzhou, China) was used to identify the species of each colony. An appraisal credibility score of >95% was considered reliable.

### 2.2. ARG Screening

All isolates were tested for ESBL and *mcr* genes via PCR (Table 1). Genomic DNA was extracted from all isolates via boiling and freeze–thawing processes, and the recovered supernatants were used as the PCR templates. The samples were screened for eight *mcr* genes (*mcr-1* to *mcr-8*) and six *bla*_ESBL_s (*bla*_TEM_, *bla*_CTX-M_, *bla*_OXA_, *bla*_C__MY_, *bla*_DHA_, and *bla*_SHV_). The PCR products were electrophoresed in 1% agarose gels and visualized under ultraviolet light (Bio-Rad, Hercules, CA, USA).

### 2.3. DNA Extraction and Genome Sequencing

DNA was extracted from the *mcr*-positive and ESBL-gene-carrying *E. coli* isolates using the Wizard Genomic DNA Extraction Kit (Promega, Madison, WI, USA). Libraries were constructed with the MGIEasy FS DNA Library Prep Set and sequenced on the MGISEQ-200RS sequencing platform (MGI). We assembled each genome using the SPAdes genome assembler (v 3.5.0). The obtained sequences were deposited in GenBank under the following BioSample numbers: SAMN25145253, SAMN25145252, SAMN25146059, SAMN25146060, SAMN25146062, SAMN25146081, SAMN25146039, SAMN25146040, SAMN25146084, SAMN25146085, SAMN25146055, SAMN25146041, SAMN25146057, SAMN25146086, SAMN25146099, SAMN25146103, and SAMN25146106.

### 2.4. Molecular Typing, Virulence Genes, ARGs, and Plasmid Identification

Multi-locus sequence type (MLST) and phylogenetic analyses were performed in silico using EnteroBase (http://enterobase.warwick.ac.uk (accessed on 24 January 2022)). Plasmid replicons and ARGs were determined in silico using online tools (http://www.genomicepidemiology.org/ (accessed on 24 January 2022)).

To reveal the possible relationships among the epidemic ESBL/*mcr-1*-carrying *E. coli* isolates, 132,786 *E. coli* genomic sequences were retrieved from GenBank, and 14,317 ESBL/*mcr-1*-carrying *E. coli* were screened. FastANI was used to compute the average nucleotide identity [20] among genomes. The top ten similar genomes obtained for each isolate sequence were selected and used to construct a maximum-likelihood (ML) tree. The coding sequences from the strains were grouped together, and a non-redundant homologous gene set was computed for the sequences using CD-HIT. We searched the homologous genes in the non-redundant homologous gene set for the coding sequences of each strain using BLAST+. If the homologous gene for a gene in the non-redundant homologous gene set existed in all selected strains and had just one copy per strain, the gene was considered a core gene. The core genes were then aligned and merged, and IQ-TREE was used to construct an ML tree.

### 2.5. Antimicrobial Susceptibility Testing

Antimicrobial susceptibility testing was performed on the *mcr*-positive and ESBL-gene-carrying *E. coli* isolates by using the reference broth microdilution method with custom plates (PRCDCN2, Thermo) for 28 antibiotics: colistin, amikacin, gentamicin, tobramycin, cefazolin, cefepime, cefoxitin, ceftazidime, ceftriaxone, cefuroxime, amoxicillin-clavulanate, ampicillin-sulbactam, piperacillin-tazobactam, aztreonam, ertapenem, imipenem, meropenem, ciprofloxacin, levofloxacin, moxifloxacin, norfloxacin, fosfomycin, tetracycline, tigecycline, minocycline, nitrofurantoin, chloramphenicol, and trimethoprim-sulfamethoxazole. The results were assessed using the CLSI (2017) breakpoints.

### 2.6. Conjugation and Transformation Analysis

The conjugation experiments were performed using *E. coli* J53 Azi^R^ (azide-resistant) as the recipients. After being incubated at 37 °C for 20 h, transconjugants were selected on Luria-Bertani agar supplemented with colistin (2 μg/mL) and sodium azide (100 μg/mL). Positive transconjugants were confirmed via real-time PCR. The transfer frequency is expressed as the number of transconjugants per total recipients.

## 3. Results

### 3.1. ESBL-Producing and Colistin-Resistant Enterobacteriaceae Isolates Screened from Tibetan Outpatients with Diarrhea

The fecal samples from the 59 outpatients were first streaked on MacConkey agar, and then suspected *E. coli* colonies were screened via real-time PCR to identify DEC isolates. In total, 18 DEC isolates were found: three EAEC, one EIEC, two EPEC, eight ETEC, and four STEC. However, none of these isolates grew on the CHROMagar ESBL, Col-apse, or SuperCARBA plates, indicating that none were ESBL-producing or colistin-resistant. Thus, these DEC isolates were distinct from the ARG-carrying isolates, suggesting that ARB and ARG monitoring should be strengthened in addition to bacterial pathogenic spectrum surveillance.

**Figure 1:**
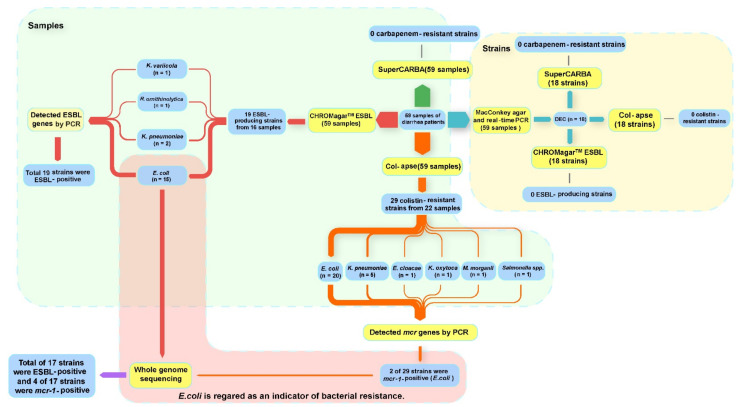
Laboratory procedure and results from examining patient fecal samples for the presence of ARB and for ARG identification.

Plating the 59 fecal samples on three types of antibiotic-containing plates yielded 48 antibiotic-resistant isolates (Figure 1). Of these, there were 19 ESBL-producing isolates (15 *E. coli*, 2 *Klebsiella pneumoniae*, 1 *Klebsiella variicola*, and 1 *Raoultella ornithinolytica*), which came from 16 of the samples (positive rate: 27.1%; Table 2). Of these ESBL-producing bacteria, 94.7% (18/19) harbored the ARG *bla*_CTX-M_, 63.2% (12/19) harbored *bla*_TEM_, and 10.5% (2/19) harbored *bla*_SHV_, but none were found to carry *bla*_DHA_ or *bla*_CMY_ genes (Table 2).

A total of 29 colistin-resistant isolates (twenty *E. coli*, five *K. pneumoniae*, one *Klebsiella oxytoca*, one *Salmonella*, one *Morganella morganii*, and one *Enterobacter cloacae*) were obtained from 22 of the 59 patients (positive rate: 37.3%) (Figure 1). Among these 29 isolates, only two *E. coli* isolates (positive rate: 6.9%) were found to harbor a gene from the *mcr* gene family; both had *mcr-1*. No carbapenem-resistant isolates were obtained from the fecal samples assessed using SuperCARBA plates (Figure 1).

### 3.2. Most ESBL-Producing and mcr-1-Carrying E. coli Isolates Were MDR and Carried ARGs

The genomes of the two *mcr*-positive *E. coli* isolates obtained from colistin plates and of the fifteen *E. coli* isolates obtained from ESBL plates were sequenced (Figure 1). Two of the *E. coli* isolates obtained from ESBL plates were found to contain *mcr-1*, as well. The *bla*_ESBL_s were also detected in both of the *mcr*-positive *E. coli* isolates that had been obtained from colistin plates.

**Figure 2:**
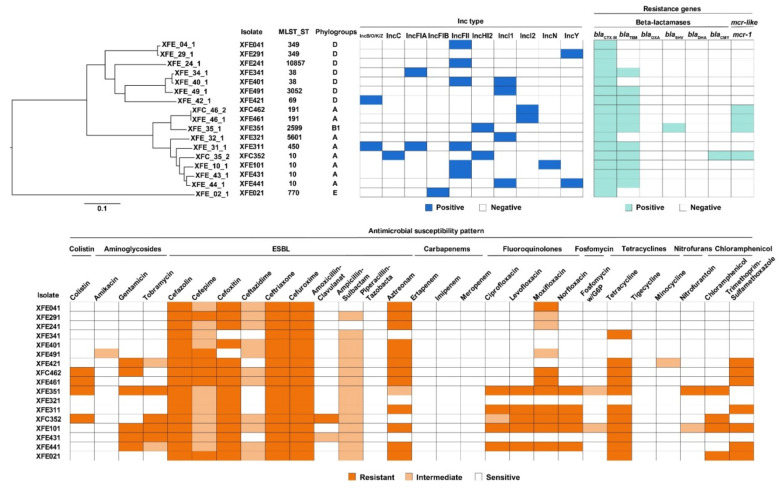
Antimicrobial susceptibility testing of 17 sequenced *E. coli* isolates. Maximum-likelihood tree (top) and MLST analysis. Tested antimicrobial susceptibilities (bottom) are presented as white, susceptible; pink, intermediate; and red, resistant.

All 17 antibiotic-resistant *E. coli* isolates were resistant to cefazolin, ceftriaxone, and cefuroxime, and >60% of these isolates were also resistant to cefoxitin, aztreonam, and tetracycline (88.2%, 70.6%, and 64.7%, respectively). All isolates were susceptible to piperacillin–tazobactam, ertapenem, imipenem, meropenem, and tigecycline. The rates of resistance to moxifloxacin and trimethoprim–sulfamethoxazole were nearly 50%. Additionally, 35.3% of isolates were resistant to gentamicin; 29.4% were resistant to levofloxacin and norfloxacin; and 23.5% were resistant to colistin, tobramycin, ciprofloxacin, and chloramphenicol. However, the isolates exhibited low rates of resistance to amoxicillin-clavulanate (5.9%) and nitrofurantoin (5.9%). In total, 10 isolates, including all four *mcr-1*-carrying *E. coli* isolates, were MDR. The isolate that was resistant to the most classes of antibiotic was resistant to seven of the tested classes (Figure 2).

**Figure 3:**
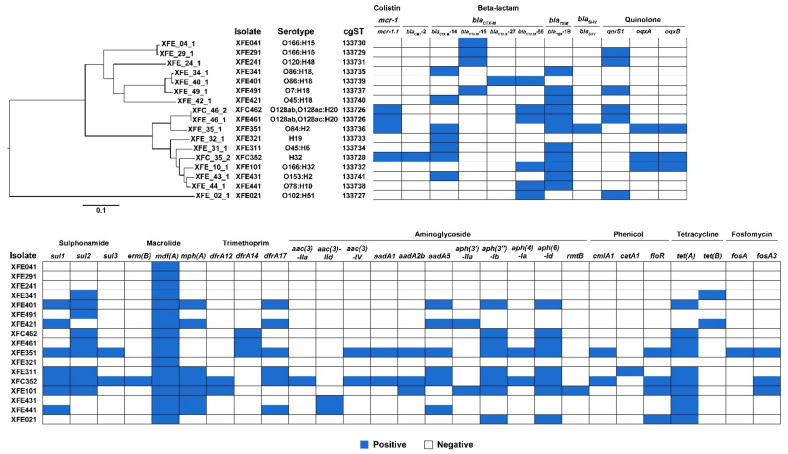
Serotype, cgMLST, and ARGs in the 17 sequenced *E. coli* isolates. Maximum-likelihood tree and MLST analysis, presence of ARGs and AR-associated plasmids; blue indicates positive.

The number of ARGs harbored by the 17 antibiotic-resistant *E. coli* isolates ranged from two to twenty-six (Figure 3). The prevalence of beta-lactam genes was 100%, with the main genotypes being *bla*_TEM-1B_ (64.7%), *bla*_CTX-M-14_ (41.2%), and *bla*_CTX-M-55_ (35.3%). The overall detection rate for tetracycline genes was 76.5% (13/17); 58.8% carried *tet(A)* and 11.8% carried *tet(B)*. The sulfonamide gene detection rate was 70.6% (12/17), with *sul2* being the predominant genotype. The aminoglycoside gene detection rate was 64.7% (11/17), with *aph(3″)-Ib* and *aph(6)-Id* predominating. The trimethoprim gene detection rate was 58.8% (10/17); *dfrA17* (35.3%), *dfrA14* (17.6%), and *dfrA12* (11.8%) were detected. Four of the 17 (23.5%) *E. coli* isolates carried the colistin resistance gene *mcr-1*. The fosfomycin gene detection rate was 17.6% (3/17). The predominant phenicol resistance gene was *floR* (23.5%). The prevalence of macrolide genes was 100%, with *mdf(A)*, *mph(A)*, and *erm(B)* being detected. The detection rate of plasmid-mediated quinolone-resistance genes was 52.9% (9/17), and these genes included *qnrS1* (35.3%), *oqxA* (17.6%), and *oqxB* (17.6%).

In total, 62 virulence genes were identified from the 17 antibiotic-resistant *E. coli* isolates. No enterotoxin or shiga-like toxin genes were found. Three isolates harbored >20 virulence genes, and *terC* was detected in all isolates; 12/17 (70.6%) isolates were positive for *iss* and *gad* genes, and 11/17 (64.7%) isolates were positive for *traT*. Only one isolate each harbored *papA_F19*, *pet*, *mcbA*, *neuC*, *kpsMII_K1*, *agg3A*, *agg3C*, *agg3D*, and *agg5A*. Three EAEC isolates (isolates XFE_32_1, XFE_43_1, and XFE_44_1) were detected (Supplementary Figure S1). Two of the three EAEC isolates harbored *bla*_CTX-M-14_, and one of the three carried *bla*_CTX-M-55_. All were resistant to cefazolin, cefoxitin, ceftriaxone, and cefuroxime.

### 3.3. All ESBL-Producing and mcr-1-Carrying E. coli Isolates Carried Plasmids, and all mcr-1 Plasmids Were Transferable

Ten incompatible plasmid types were found: IncFII (6 isolates; 60.0%), IncI1 (4 isolates; 40.0%), IncB/O/K/Z (2 isolates; 20.0%), IncFIA (2 isolates; 20.0%), IncHI2 (2 isolates; 20.0%), IncI2 (2 isolates; 20.0%), IncY (2 isolates; 20.0%), IncC (1 isolate; 10.0%), IncFIB (1 isolate; 10.0%), and IncN (1 isolate; 10.0%). All of the ESBL-producing and *mcr-1*-carrying *E. coli* isolates harbored plasmids, and five harbored more than one plasmid. Isolate XFE_31_1 harbored three plasmids: IncB/O/K/Z, IncFIA, and IncFII. Four isolates carried two plasmids each (23.5%, 4/17). Two distinct *E. coli* isolates from a single patient each carried IncHI2, and both carried *mcr-1*, further confirming that *mcr-1* can be transmitted horizontally between bacteria through IncHI2 in vivo.

Conjugation experiments revealed that all four *mcr*-harboring *E. coli* isolates could transfer their *mcr*-carrying plasmids to *E. coli* J53, but they did so with low transfer frequencies (~10^−6^ colony-forming units/donor).

### 3.4. ESBL-Producing and mcr-1-Carrying E. coli Isolates from Outpatients in Lhasa Had Clonality with Strains from Other Regions and Countries

The 17 antibiotic-resistant *E. coli* isolates sequenced here were found to belong to 11 sequence types (STs; Figure 2): ST10 (n = 4), ST38 (n = 2), ST69 (n = 1), ST191 (n = 2), ST349 (n = 2), ST450 (n = 1), ST770 (n = 1), ST2599 (n = 1), ST3052 (n = 1), ST5601 (n = 1), and ST10857 (n = 1). The most prevalent ST was ST10, found in isolates from four patients. Core-genome (cg) MLST revealed that these 17 antibiotic-resistant *E. coli* isolates belonged to 16 distinct cgSTs; only isolates XFE_46_1 and XFC_46_2 had the same cgST. Most of the 17 antibiotic-resistant *E. coli* isolates belonged to phylogroup A, with the next most prevalent phylogroups being D, B1, and E.

**Figure 4:**
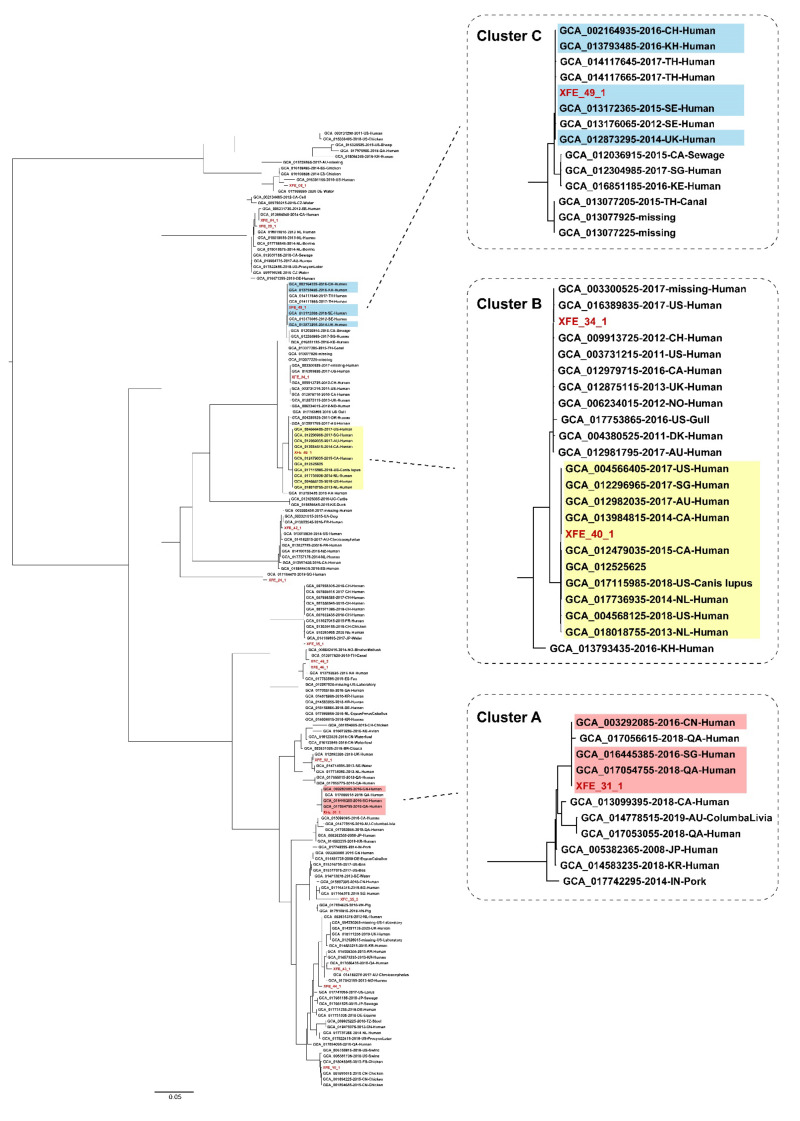
Phylogenetic analysis of the 17 antibiotic-resistant *E. coli* isolates conducted with sequences from GenBank. The phylogenetic analysis was conducted on the 17 antibiotic-resistant *E. coli* isolates from the study outpatients and another 170 sequences of *bla*_CTX-M-14/15/27/55_- or *mcr-1*-carrying *E. coli* retrieved from GenBank. The strains in each of the three clonal clusters (Clusters A, B, and C), which each had <19 core SNPs, are highlighted in color. IE: Ireland; EE: Estonia; SE: Sweden; AU: Australia; BR: Brazil; DK: Denmark; DE: Germany; FR: France; CO: Colombia; KR: Korea; NL: Netherlands; CA: Canada; KH: Cambodia; CZ: Czech Republic; QA: Qatar; UG: Uganda; UA: Ukraine; KE: Kenya; US: United States; NO: Norway; JP: Japan; TH: Thailand; ES: Spain; SG: Singapore; IN: India; NE: Niger; CH: Switzerland; VN: Vietnam; UK: United Kingdom; CN: China.

To evaluate the genomic clonality and similarity of the 17 antibiotic-resistant *E. coli* isolates, their phylogenetic relationships were analyzed based on recombination-free single nucleotide polymorphisms (SNPs; Figure 4). Isolates XFC_46_2 and XFE_46_1 were obtained from different plates of the same sample, but no core SNPs existed in their chromosome genomes or had the same ARG composition patterns, strongly suggesting that isolates XFC_46_2 and XFE_46_1 were from the same strain. However, isolates XFC_35_2 and XFE_35_1, which were also obtained from different plates of the same sample, were found to belong to distinct phylogenetic branches, suggesting that isolates XFC_35_2 and XFE_35_1 were distinct strains.

To evaluate the genomic clonality and similarity of the 17 antibiotic-resistant *E. coli* isolates with strains from other regions and countries, we first retrieved 132,786 *E. coli* genomic sequences from GenBank and selected 14,317 *bla*_CTX-M-14/15/27/55_ and *mcr-1*-carrying *E. coli* genomes that had been blasted with *bla*_CTX-M-14/15/27/55_ and *mcr* family genes. For each sequence obtained, we selected the top ten similar genomes and used them to construct an ML tree. At least three clonal clusters (clusters A, B, and C; Figure 4) of strains were identified. In each cluster, a limited number of core SNPs (11–19) existed in the chromosomal genomes, which were each separated from the nearest neighbor isolate by >30 core SNPs. In each cluster, the strains with similarity were isolated from different regions/countries or in different collection years. This finding may suggest the broad spread of some antibiotic-resistant *E. coli* strains. Among these similar strains, strains GCA_003292085 (14 SNPs with isolate XFE_31_1) and GCA_002164935 (19 SNPs with isolate XFE_49_1) were isolated from China in 2016. Specifically, the similarity of one study isolate with strain GCA_002164935, isolated from Sichuan Province, which borders Tibet and has significant trade exchanges with Tibet, may suggest the spread of this strain through food trade or human travel throughout different provinces in China.

## 4. Discussion

In this study, we screened fecal samples from 59 Tibetan outpatients with diarrhea for ARB and detected the ARGs harbored by these isolates. The fecal samples of 16 patients contained ESBL-producing bacteria, and the fecal samples of 22 patients had polymyxin-resistant bacteria. We found high rates of AR, suggesting that AR spread and food hygiene are problems in Tibet. Antibiotic-resistant bacteria can spread across borders via trade and travel, and the global trade of food may rapidly spread MDR bacteria between cities and countries. *E. coli*, a commensal species and opportunistic pathogen commonly found in the intestinal tracts of animals and humans [27], is regarded as an indicator organism of AR for many bacteria [28]. Global food trade may increase the spread of ESBL-producing *E*. *coli* [29]. The urbanization of Tibet is accelerating, strengthening communication between its cities, such as Lhasa, and the rest of China. The *E*. *coli* isolates obtained from Lhasa in this study were found to be closely related to those isolated from Sichuan Province, which borders Tibet, as well as to strains isolated from other countries. Sichuan and Tibet, both in Western China, have many shared commodities and personnel interactions, which could increase the spread of ARB and ARGs. An investigation showed that diarrhea was a risk factor for acquiring ESBL-producing Enterobacteriaceae during international travel [30].

Most of the antibiotic-resistant *E. coli* isolates from this study belonged to phylogroups A (47.1%) and D (41.2%). A previous study reported that most *E. coli* isolated from yaks in Tibet also belonged to phylogroup A [31]. Several clusters with similar chromosomes were identified, strongly suggesting that the rapid expansion of ESBL-producing strains in the community presents a potential public health threat. Active surveillance may help prevent their expansion. Agricultural animals have been singled out as the most likely reservoirs for the amplification and spread of antibiotic-resistant Enterobacteriaceae. Residual antibiotics in the environment increase the selective pressure incurred by ARB and stimulate ARG transfer. ARB and ARGs transmitted in the environment pose risks to human and animal health.

All 17 ARB isolates obtained from the fecal samples of patients with diarrhea were resistant to cefazolin, ceftriaxone, and cefuroxime. Previous studies reported that *E. coli* carried by yaks in Tibet had high rates of MDR [9,31]. In this study, 10 of the *E. coli* isolates were MDR, and most were resistant to seven types of antibiotics. The results of this study indicate that the issue of AR among Tibetans living in Lhasa City is very serious. Fortunately, no carbapenem-resistant bacteria were isolated from Tibetans with diarrhea.

In this study, the most common ARGs were *bla*_TEM-1B_, *bla*_CTX-M-14_, *bla*_CTX-M-55_, and *bla*_CTX-M-15_, and the ARG with the highest prevalence was *bla*_CTX-M_. These results are the same as those from previous studies, which indicate that *bla*_CTX-M_ has been the main *bla*_ESBL_s disseminated worldwide [32,33]. Additionally, *bla*_CTX-M-15_ and *bla*_CTX-M-14_ have been reported to be the most important *bla*_ESBL_s in humans, animals, and the environment worldwide [34,35], and our findings of relatively high carriage rates of both *bla*_CTX-M-14_ and *bla*_CTX-M-15_ echo this. A nationwide county hospital survey found that the incidence of *bla*_CTX-M-55_ exceeded that of *bla*_CTX-M-15_ in China [36]. Our findings indicate that, among Tibetan outpatients with diarrhea, the percentage of isolated bacteria carrying *bla*_CTX-M-55_ is relatively high.

We detected no ESBL-producing or *mcr*-carrying ETEC or STEC in our study, but we isolated three ESBL-producing EAEC. EAEC strains are among the opportunistic pathotypes harboring the virulence-determining *aggR* [37]. Two of the three EAEC strains were *bla*_CTX-M-14_-positive, and 79% of 29 EAEC strains from patients with diarrhea in Japan were *bla*_CTX-M-14_-positive [38], suggesting that clinicians should estimate the threat of ESBL-gene-harboring EAEC in community-acquired infections. Although the pathogenic strain may lack ARGs, other intestinal bacteria from the same individual may carry ARGs, especially plasmid-borne ARGs, and these ARGs may be transferred among intestinal bacteria. In addition to pathogenic spectrum surveillance, AMR surveillance should include ARB and ARG monitoring in common intestine-colonizing bacteria.

In this study, four *mcr-1*-carrying *E. coli* isolates (6.7%) were obtained from fecal samples of Tibetan outpatients with diarrhea. In China, among 8022 fecal samples collected from patients at three hospitals in Guangzhou, 497 (6.2%) were *mcr-1*-positive [39]; the carriage rate of *mcr-1* for Tibetans in the present study was similar. Among the four *mcr-1*-carrying *E. coli* isolates, three belonged to phylogroup A, and one belonged to phylogroup B1, a result identical to that of a previous study in Guangzhou, China [40]. Two plasmids (IncHI2 and IncI2) were found in our *mcr-1*-carrying isolates; these are the same plasmid types that were found in *mcr-1*-harboring *Salmonella* from outpatients with diarrhea in Shanghai, China [17]. IncHI2 is a large *mcr-1*-carrying plasmid that is common worldwide [41]. This plasmid has been associated with *E. coli* recovered from different sources in China and is ubiquitous in different hosts. Plasticity and the ability to acquire different ARGs, and potentially IncHI2, may be responsible for disseminating these ARGs worldwide in different bacterial species [42]. In our study, two distinct *E. coli* isolates from one patient each contained an *mcr-1*-positive IncHI2, indicating that IncHI2 may be transmitted horizontally between bacteria within human intestines.

In summary, Tibet, which is considered to have minimal antibiotic pollution, is seriously threatened by ARB and ARGs. The Tibetan outpatients with diarrhea carried high numbers of ARB, which may have spread from other provinces or countries via food commerce or travel, indicating that Tibetans are confronted with severe AR. Within the framework of a One Health approach, actions to minimize and mitigate the spread of ARB should be implemented in Tibet. Surveillance, in addition to bacterial pathogenic spectrum monitoring, is required to monitor ARB and ARG prevalence and transmission in humans, animals, and the environment to better understand the potential threat of ARB and ARGs to public health.

## Figures and Tables

**Table 1:** Primers used for PCR.

Target Gene	Primers	Sequences of Primers (5′ to 3′)
*mcr-1* [17]	mcr-1-F	TCGGCTTTGTGCTGACGAT
	mcr-1-R	AAATCAACACAGGCTTTAGCACATA
	mcr-1-P	(FAM)CTGTCGTGCTCTTTG(MGB)
*bla*_TEM_ [18]	blaTEM-F	GCATCTTACGGATGGCATGA
	blaTEM-R	CCTCCGATCGTTGTCAGAAGT
	blaTEM-P	ATTATGCAGTGCTGCCATA ACCATGA
*mcr-2* [19]	mcr-2-F	AGCCGAGTCTAAGGACTTGATGAATTTG
	mcr-2-R	GCGGTATCGACATCATAGTCATCTTG
*mcr-3* [19]	mcr-3-F	CCAATCAAAATGAGGCGTTAGCATAT
	mcr-3-R	TGAGCAATTTCACTATCGAGGTCTTG
*mcr-4* [20]	mcr-4-F	TCACTTTCATCACTGCGTTG
	mcr-4-R	TTGGTCCATGACTACCAATG
*mcr-5* [21]	mcr-5-F	ACTCGACTGCCACCAGATCATCG
	mcr-5-R	CGCTGGAGTGTCAAGCCACTACTG
*mcr-6* [22]	mcr-6-F	GTCCGGTCAATCCCTATCTGT
	mcr-6-R	ATCACGGGATTGACATAGCTAC
*mcr-7* [22]	mcr-7-F	TGCTCAAGCCCTTCTTTTCGT
	mcr-7-R	TTCATCTGCGCCACCTCGT
*mcr-8* [22]	mcr-8-F	AACCGCCAGAGCACAGAATT
	mcr-8-R	TTCCCCCAGCGATTCTCCAT
*bla*_CTX-M_ [23]	blaCTX-M-F	TTT GCG ATG TGC AGT ACC AGT AA
	blaCTX-M-R	CGA TAT CGT TGG TGG TGC CAT A
*bla*_OXA_ [24]	blaOXA-F	GGC ACC AGA TTC AAC TTT CAA G
	blaOXA-R	GAC CCC AAG TTT CCT GTA AGT G
*bla*_SHV_ [25]	blaSHV-F	TTA TCT CCC TGT TAG CCA CC
	blaSHV-R	GAT TTG CTG ATT TCG CTC GG
*bla*_CMY_ [26]	blaCMY-F	GAC AGC CTC TTT CTC CAC A
	blaCMY-R	TGG AAC GAA GGC TAC GTA
*bla*_DHA_ [26]	blaDHA-F	CTG ATG AAA AAA TCG TTA TC
	blaDHA-R	ATT CCA GTG CAC TCC AAA ATA

**Table 2:** Enterobacteriaceae isolated from chromogenic media.

Source		Species		Genes Detected by PCR	
Plate Type	No. of Strains	Species of Strains	No. of Strains	Resistant Genes or Genetic Elements Studied	No. of Strains
ESBLs	19	*Escherichia coli*	15	*bla*_CTX-M_	6
				*bla*_CTX-M_ + *bla*_TEM_	7
				*bla*_CTX-M_ + *bla*_TEM_ + *bla*_SHV_	1
		*Klebsiella pneumoniae*	2	*bla*_CTX-M_ + *bla*_TEM_	1
				*bla*_CTX-M_ + *bla*_TEM_ + *bla*_SHV_	1
		*Klebsiella variicola*	1	*bla*_CTX-M_	1
		*Raoultella ornithinolytica*	1	*bla*_CTX-M_ + *bla*_CYM_	1
Colistin	29	*Enterobacter cloacae*	1		
		*Escherichia coli*	20	*mcr-1*	2
		*Klebsiella oxytoca*	1		
		*Klebsiella pneumoniae*	5		
		*Morganella morganii*	1		
		*Salmonella* spp.	1		

## Data Availability

The datasets used and/or analyzed during the current study are available from the corresponding author up on reasonable request.

## Supplementary Materials

The following supporting information can be downloaded at https://www.mdpi.com/article/10.3390/antibiotics11040508/s1. Figure S1: Characteristics of the virulence genes predicted based on the sequences of the 17 sequenced *E. coli* isolates.
