# Supplementary material for: High Carriage of Extended-Spectrum, Beta Lactamase-Producing, and Colistin-Resistant Enterobacteriaceae in Tibetan Outpatients with Diarrhea
Source: Antibiotics (Basel). 2022 Apr 11;11(4):508. doi: 10.3390/antibiotics11040508 (PMC9032258; doi:10.3390/antibiotics11040508)
Supplement: Supplementary file 1 [file antibiotics-1614080-supplementary.pdf › antibiotics-1614080-supplementary.pdf]

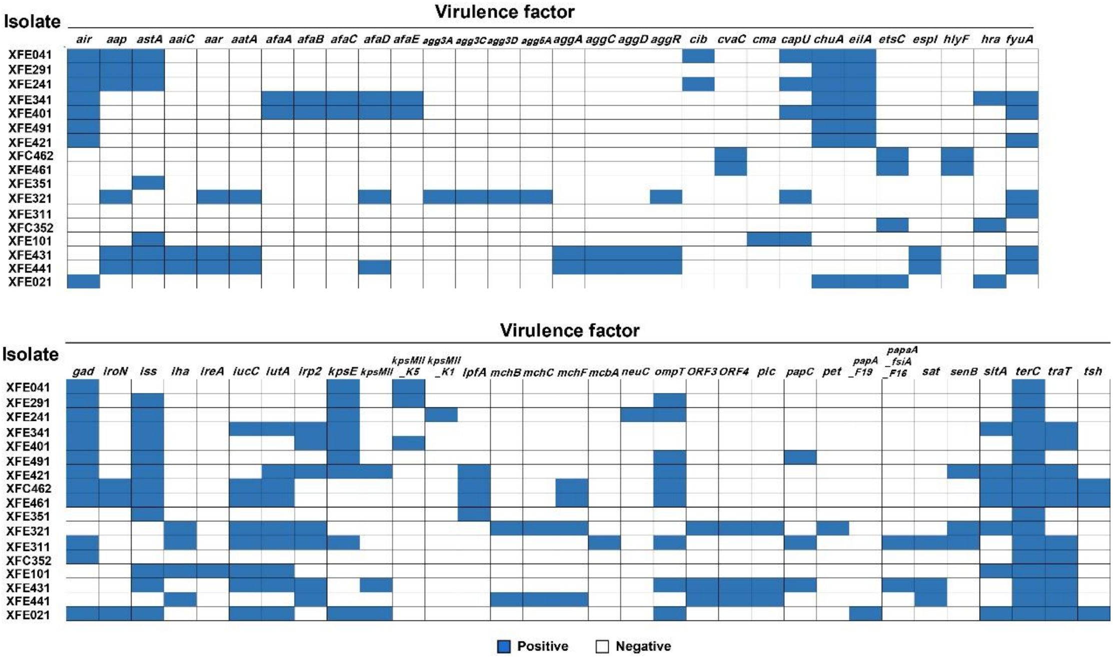


**Supplementary Figure S1**. Characteristics of the virulence genes predicted based on the sequences of the 17 sequenced *E. coli* isolates.
